# Dietary Therapies in Inflammatory Bowel Disease and Their Effects on Disease Activity and the Gut Microbiome

**DOI:** 10.3390/nu18142240

**Published:** 2026-07-09

**Authors:** Gila Sasson, Caitlin Hosmer, Joshua Korzenik

**Affiliations:** Division of Gastroenterology, Hepatology and Endoscopy, Brigham and Women’s Hospital, Harvard Medical School, Boston, MA 02115, USA

**Keywords:** dietary interventions, inflammatory bowel disease (IBD), Crohn’s disease, ulcerative colitis, microbiome, enteral nutrition, Mediterranean diet, nutritional therapy

## Abstract

The rapid rise in inflammatory bowel disease (IBD) worldwide parallels urbanization and Westernization, including a shift towards the Western diet. This evolving epidemiological landscape shines a spotlight on the contributions of the environment to IBD pathogenesis and has generated particular interest in the role of diet as both a therapeutic and preventative strategy. Although epidemiologic studies have identified dietary risk associations and dietary intervention studies have demonstrated symptomatic benefit, the specific dietary components that influence disease course and the complex mechanistic pathways through which they act are incompletely understood. In this narrative review, we examine the clinical efficacy of dietary therapies studied in IBD and discuss their effects on gut microbial composition and function, recognizing the heterogeneity of evidence across dietary approaches and the evolving nature of this field. We also discuss emerging evidence linking diet, microbial metabolism and immune response, and consider how a better understanding of these interactions may inform future therapeutic strategies, optimize dietary interventions, and support the development of precision nutrition approaches in IBD. Overall, current evidence suggests that dietary therapies may benefit selected patients with IBD and are associated with changes in the gut microbiome, although their mechanisms and optimal clinical application require further study.

## 1. Introduction

**Figure 1:**
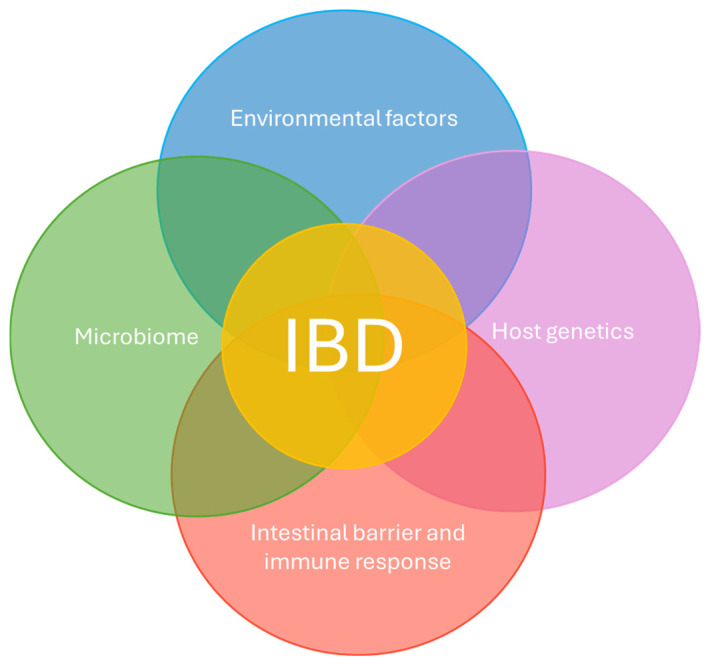
Current understanding of IBD pathogenesis: multidimensional interactions among key domains.

Inflammatory bowel disease (IBD) is a chronic relapsing–remitting inflammatory disorder of the gastrointestinal tract, with the predominant phenotypes being ulcerative colitis (UC) and Crohn’s disease (CD). Although the pathogenesis of IBD continues to be explored, it is widely understood that it arises from complex interactions between environmental variables and the immune response mediated by the gut microbiome in genetically susceptible hosts (Figure 1). The global acceleration of IBD incidence, in countries beyond North America and Western Europe, arising in parallel with industrialization and Westernization, underscores the significant role of environmental influences in IBD pathogenesis [1].

Diet is felt to be a predominant environmental variable that influences the risk of developing IBD as well as the course of established disease. Epidemiological studies have shown that a Western-like dietary pattern, rich in animal fats and proteins and refined sugar, and low in fruits, vegetables, and vitamin D, is associated with an increased risk of developing IBD [1,2]. In contrast, a diet rich in fruits, vegetables, and dietary fibre is associated with a reduced risk of IBD [1]. As a result, dietary optimization provides a therapeutic opportunity in IBD, either alone or in conjunction with other therapies.

Specific dietary components, as well as broader dietary patterns, can modulate the composition and function of the gut microbiome. Habitual dietary patterns can shape microbial composition into defined community structures known as enterotypes over the long term [3]. For example, healthy children in a rural village in Africa who consumed a predominantly plant-based habitual diet, enriched in polysaccharides and fibre, had a distinct microbial signature that was starkly different than that of children in a European city who consumed a Western-like habitual diet, emphasizing animal protein, sugar, and fat [4]. However, substantial short-term dietary modification can also induce taxonomic shifts. A controlled feeding study found that drastic dietary change between strictly animal-based and plant-based diets led to microbial alteration in as little as one day, which was subsequently reversed after two days of diet cessation [5].

In addition to specific nutritional components, such as types and profiles of macronutrients, other aspects of diet may impact health in IBD. Food additives, including emulsifiers, have been a growing area of interest, with animal data supportive of a role in damaging the intestinal barrier and contributing to inflammation. Highly processed foods have also been implicated in worsening IBD, and diets avoiding those components may offer potential benefits.

Given the complex relationship between diet, microbiome, immune regulation, and the development and progression of IBD, it is becoming clear that diet is a potential vehicle for meaningful intervention in IBD. Although efforts are being made to develop microbial-directed therapies for IBD, including prebiotics, probiotics, antibiotics, and fecal transplant, preserving and augmenting microbial change requires ongoing maintenance of such therapies [6]. Dietary therapies that emphasize beneficial substances while reducing harmful ones can modulate the gut microbiome and host immune response. With appropriate nutritional guidance, these diets may be able to be maintained long term. A variety of diets have been proposed as therapy for IBD to improve symptoms, however their effects on disease activity are less clear and require further study to support high-quality evidence-based recommendations. Understanding the effects of both individual dietary components and broader dietary patterns is also important, although this is a formidable endeavour given the multitude of possible dietary exposures and their complex interactions with the gut microbiome and host immune response. Data emerging from clinical and preclinical studies involving the microbiome, metabolome and inflammatory cascade are beginning to shed light on the mechanistic pathways through which dietary interventions influence IBD onset and progression. These insights may help to further elucidate IBD pathogenesis and guide targeted dietary therapies going forward.

With the growing interest in diet as therapy for UC and CD, whether as primary therapy or adjunctive treatment, the number of dietary trials in IBD has been increasing despite the challenges in conducting them. The call from people living with IBD has prompted a renewed interest in dietary interventions [6]. In addition, the nascent effort to identify early interventions that may delay disease progression or even prevent disease onset in those at high risk has further expanded the focus on diet. A central premise underlying these efforts is that dietary interventions may influence disease activity through modulation of the gut microbiome and its downstream effects on intestinal inflammation. However, existing data often remain dispersed across individual interventions, with clinical and microbiome outcomes not always considered together, and symptomatic improvement not necessarily reflecting underlying inflammatory activity.

To examine the interactions between diet, the gut microbiome and disease activity in IBD, a literature search was conducted in PubMed using the key terms “inflammatory bowel disease”, “IBD”, “Crohn’s disease”, “ulcerative colitis”, “nutritional therapy”, “diet”, “microbiome”, and “(partial) enteral nutrition”. Additional studies were identified through manual review of the reference lists of retrieved articles. As this is a review, article selection was guided by topical relevance and scope rather than predefined inclusion or exclusion criteria, with emphasis placed on clinical studies of dietary interventions in IBD, investigations of diet–microbiome–immune interactions, and key review articles providing contextual and mechanistic insights.

This review aims to integrate current evidence on the clinical efficacy of dietary therapies in IBD, with a particular focus on their effects on disease activity and the gut microbiome. We provide a comparative synthesis of the efficacy, limitations and clinical applicability of these dietary approaches, and highlight key knowledge gaps and emerging directions in precision nutrition that may inform more individualized dietary strategies in IBD.

## 2. Exclusive Enteral Nutrition

Exclusive enteral nutrition (EEN) is the most widely studied dietary intervention in CD, and it is currently first-line therapy for induction of remission in mild-to-moderate active CD in the European Crohn’s Colitis Organization and European Society of Pediatric Gastroenterology, Hepatology and Nutrition consensus guideline recommendations [7,8]. EEN was originally implemented as adjunct therapy in the pre-operative setting to optimize nutritional fitness in adult and pediatric patients with CD complicated by malnutrition [9]. Its anti-inflammatory potential was realized following observations that EEN led to improved disease activity whereby some patients no longer required surgery [9]. It has since been shown in a number of studies to be equivalent if not superior to steroids in inducing remission in CD.

### 2.1. Diet Regimen

EEN is a formula-only diet using an elemental, semi-elemental, or polymeric formula (Table 1). Notably, its therapeutic efficacy does not appear to depend on its nitrogen source, given that various formulas have been studied with no significant differences in efficacy observed between them [10,11,12,13,14]. Formulas varying in lipid composition have also been studied, with no differences in efficacy observed [14]. This knowledge is useful on both a practical and mechanistic level. From a practical perspective, polymeric formulas are more palatable and can be administered orally, while elemental formulas are less palatable and are therefore often administered via nasogastric tube. As a result, polymeric formulas are generally first line in order to enhance adherence, while elemental formulas are predominantly restricted to individuals with intolerance to cow’s milk protein [9]. The treatment duration in clinical studies has ranged between 1 and 12 weeks, though the majority of studies and IBD centres have routinely prescribed 6–8 weeks.

### 2.2. Effects on Disease Activity

A number of studies, including randomized controlled trials (RCTs), have demonstrated EEN to be as effective as or superior to corticosteroids in inducing clinical remission and mucosal healing in pediatric CD, with remission rates of approximately 80% [2,15,16,17,18,19,20,21,22,23] (Table 2).

A meta-analysis of eight studies comparing EEN and corticosteroids in a total of 451 pediatric patients demonstrated that EEN was as effective as steroids in inducing remission (OR = 1.26 [95% CI 0.77, 2.05] and superior to steroids in achieving mucosal healing (OR = 4.5 [95% CI 1.64, 12.32]) [29]. EEN’s efficacy in improving disease activity was further demonstrated in a North American prospective cohort study in pediatric CD patients that compared EEN with anti-TNF therapy [30,31]. EEN induction studies in pediatric patients have also demonstrated its ability to reduce, though generally not normalize, fecal calprotectin (FCP) by 8 weeks, particularly in those who achieve clinical remission [9,13,32]. This suggests that longer courses of EEN may be beneficial [9,33].

In contrast to EEN’s high success rate in pediatric CD, the adult experience has been less favourable. A meta-analysis of eight trials (*n* = 409 participants) demonstrated EEN to be inferior to steroids in inducing clinical remission in adults (RR 0.65, 95% CI 0.52 to 0.82) [33]. This finding may be explained by the generally high discontinuation rate in adult EEN studies, which is largely due to the monotony and poor palatability of EEN [9]. It also buttresses the notion that EEN is most effective when delivered in IBD centres of excellence, with multidisciplinary care teams who have the resources and experience to best support the regimen [9,34].

Whereas EEN was initially thought to be most effective in small bowel CD, with some studies demonstrating improved mucosal healing with ileal compared to colonic involvement, later studies have demonstrated its efficacy regardless of luminal disease distribution [9,35]. Its efficacy in isolated oral or perianal CD has not been consistently demonstrated, and its use in penetrating CD is unclear [9].

Evidence for supporting the use of EEN in UC is limited and largely restricted to its use as adjunctive therapy. In a recent open-label RCT of 62 adult patients with acute severe UC, EEN combined with corticosteroids was associated with lower corticosteroid failure rates (intention-to-treat [ITT] analysis 25% vs. 43%, *p* = 0.051; per protocol analysis 19% vs. 43%, *p* = 0.04), shorter hospitalizations (median 10 vs. 13 days; *p* = 0.04), greater reductions in C-reactive protein (CRP) and FCP (both *p* = 0.04), and lower rates of composite colectomy/hospitalization at 6 months (16% vs. 39%; *p* = 0.045) compared with corticosteroids alone [36].

### 2.3. Mechanisms of Action and Effects on the Gut Microbiome

While the specific mechanisms of action of EEN remain unclear, it is widely believed to involve several pathways, including influencing host immune response, optimizing intestinal permeability, and modulating the gut microbiome [37]. It is also understood that the exclusion of additional foods is crucial to its efficacy rather than the specific composition of the formula [9]. This is supported by EEN’s superiority over partial enteral nutrition with an unrestricted diet in inducing clinical remission and improving serologic inflammatory markers in CD [38]. While the overall nutritional composition of EEN includes anti-inflammatory properties such as limited food allergens, absence of nucleotides, lack of additional additives, and an anti-inflammatory lipid profile, there is mounting evidence that EEN also exerts its benefits via modulation of the gut microbiome [9]. However, there are limited studies evaluating the effects of EEN on the gut microbiome (Table 3).

This challenge is compounded by heterogeneity in patient populations, methodologies, formula types, and incorporation or omission of oral foods [51]. Despite this heterogeneity, greater taxonomic shifts and decreased bacterial diversity have consistently been observed in CD patients compared to healthy controls, and often correlate with clinical improvement [23,39,40,51,52]. Initially, small studies reported significant decreases in the abundance of *Bacteroides*/*Prevotella* during EEN, as well as a correlation between *Bacteroides*/*Prevotella* abundance and Pediatric Crohn’s Disease Activity Index (PCDAI) [39,40,51]. This resonates with a study in HLA-B27 transgenic rats, that found that colonization with Bacteroides was associated with luminal inflammation [53]. Altogether, this suggests a potential association between *Bacteroides* spp. and CD, which may be modulated by EEN. Additional shifts within the Bacteroidetes phylum include reductions in members of the families *Bacteroidaceae*, *Porphyromonadaceae* and *Rikenellaceae* [39,40,41,51].

Studies have also demonstrated a variable response within the Firmicutes phylum to EEN. Small studies in pediatric CD observed decreased abundance in six families (*Erysipelotrichaceae*, *Ruminococcaceae*, *Lachnospiraceae*, *Streptococcaceae*, *Veillonellaceae*, and *Peptostreptococcaceae*) as well as *Clostridium coccoides*, and increased abundance in members of the *Christensenellaceae* family during EEN, which generally correlated with clinical improvement [23,39,41]. Reduction in butyrate-producing *Faecalibacterium prausnitzii* spp. has also been reported, which likely reflects the lack of fermentable fibre in EEN and implies that *F. prausnitzii* has little influence on the therapeutic efficacy of EEN [32,42,51].

One study demonstrated decreases in both taxonomic and functional diversity during EEN [43]. Of 34 genera that changed significantly, most decreased in relative abundance with the most negatively impacted, including *Ruminococcus* and commensal organisms *Bifidobacterium*, and *Faecalibacterium. Lactococcus* was the only genus that significantly increased. There was an increase in genes encoding for spermidine/putrescine (*p* = 0.031) and the shikimate pathway (*p* = 0.058), which are involved in cell growth and may reflect epithelial repair. In contrast, there was a reduction in genes involved in the biosynthesis of B-complex vitamins biotin (*p* = 0.005) and thiamine (*p* = 0.017), which may reflect a decrease in the bacteria possessing these genes or alterations in fatty acid synthesis involving these vitamins. Larger intervention studies are needed to elucidate whether these findings play a role in EEN’s therapeutic mechanism of action.

The microbiome may also be of value in predicting response to EEN. In a metagenomic analysis of pediatric CD patients, Lewis et al. observed a significant difference in microbial composition that emerged between ultimate responders and non-responders after one week, suggesting that the microbiome may play a role in early prediction of EEN response [54]. By 8 weeks, EEN responders had microbial compositions more closely resembling those of healthy controls while non-responders were more dissimilar [51,54]. More recently, pre-treatment microbial and metabolomic signatures were explored as possible predictors of EEN response in a prospective cohort study of pediatric CD patients [55]. EEN responders had increased microbial richness (*p* = 0.015) and reduced butyrate (*p* = 0.03), acetate (*p* = 0.027), and phenylacetate (*p* = 0.021) compared to non-responders. In contrast, non-responders had increased abundance of *Ruminococcaceae*, *Lachnospiraceae* and *Bacteroides*, and increased concentrations of butyrate, acetate and phenylacetate. Although observational in design and fairly small in sample size, these studies suggest the potential for future dietary personalization approaches using noninvasive, readily available tools.

In addition to alterations in the microbiome as above, there are likely additional mechanistic pathways through which EEN confers benefit, both directly and indirectly. Its effects may arise not only from what is being ingested but also what is not being consumed, such as highly processed foods, gluten, dairy, emulsifiers and other food additives.

### 2.4. Summary

The body of literature on EEN, inclusive of high-quality studies, has established its favourable efficacy in inducing clinical remission in CD and that it is comparable to corticosteroids [8]. It is recommended as first-line induction therapy in pediatric CD patients with mild-to-moderate disease activity, whereas in adults it is primarily used as second- or third-line therapy, adjunctive therapy or preoperative optimization, particularly in complex and/or refractory disease, in part due to issues of tolerability and adherence [8]. Further, a recent RCT found that combination therapy with EEN and corticosteroids is superior to corticosteroids alone in inducing remission in UC. Small studies have also demonstrated its efficacy in mucosal healing however this requires further corroboration in high-quality studies. Added benefits of EEN include avoidance of corticosteroids in CD and correction of nutritional deficiencies, while some drawbacks are poor palatability and dietary monotony which make it a less pragmatic approach for adults.

A number of microbial changes observed during EEN appear counterintuitive and may reflect several contributing factors, including the nutritional composition of EEN formulas, the exclusion of habitual diet, and modulation of intestinal inflammation. Accordingly, whether these microbial alterations directly contribute to clinical improvement or represent downstream effects remains uncertain. To that end, the notion of modifying nutritional composition to stimulate growth of beneficial taxa suppressed during EEN is an interesting avenue to explore [43].

## 3. Partial Enteral Nutrition

While the therapeutic efficacy of EEN has been demonstrated for induction of remission, particularly in the pediatric population, it is a challenging regimen to continue long term due to difficulties with tolerance and the psychosocial toll it may exert [56]. As such, the literature is limited around its use as maintenance therapy [57]. Instead, there has been increasing interest in partial enteral nutrition (PEN) as maintenance therapy in quiescent disease, which consists of an elemental formula diet delivered in combination with an oral diet. The formula is meant to provide a percentage of daily caloric requirements, while the oral diet can be exclusionary or unrestricted [34,56].

### 3.1. Effects on Disease Activity

PEN has demonstrated potential for both induction and maintenance of remission of CD however, like EEN, there is heterogeneity among studies and outcomes have varied [58]. The heterogeneity may predominantly be due to lack of standardization of the composition and daily caloric allowance of the oral diet, as well as variation in treatment duration [34,56]. A systematic review of controlled trials-comprising 307 adult and pediatric CD patients- assessed PEN’s efficacy in inducing clinical remission and found high rates of symptomatic response to both EEN and PEN, with no significant differences between diets [56]. FCP response was mixed and generally aligned with the oral diet component of PEN, with an increase in FCP observed following re-introduction of an unrestricted oral diet versus a sustained response on an exclusion diet, such as the CDED. This was similarly observed in a prospective study of 90 children with active CD that compared PEN with an unrestricted diet, EEN, and anti-TNF therapy, which found PEN to be less efficacious in reducing mucosal inflammation (FCP ≤ 250 μg/g: PEN 14%, EEN 45%, anti-TNF 62%, *p* = 0.001) [30]. In contrast, Urlep et al.’s prospective cohort study of 25 pediatric patients with CD compared clinical and mucosal response to EEN (*n* = 13) and PEN, with 75% of caloric needs provided from a polymeric formula plus one daily meal from an anti-inflammatory diet (*n* = 12) over 6 weeks [59]. ITT analysis found no difference in symptomatic and endoscopic remission rates between the two arms. These findings support the hypothesis that the therapeutic potential of diet is rooted in excluding certain dietary components as opposed to including others.

PEN has also been used effectively to maintain clinical remission in CD [60]. A recent meta-analysis of prospective studies comprising 429 CD patients demonstrated superiority of PEN in preventing clinical relapse for up to 2 years (RR 0.67, 95% CI: 0.54–0.82, *p* < 0.01) and maintaining clinical remission for up to 1 year (RR 1.32, 95% CI: 1.07–1.64; *p* = 0.01) compared to no dietary therapy [57]. In one prospective study of 39 adult CD patients in clinical remission, ITT analysis found that PEN with an unrestricted oral diet (*n* = 21) was associated with increased remission rates at 12 months compared to a standard unrestricted diet (*n* = 18) (48% vs. 22%, *p* < 0.0003) [61]. This was further studied in a RCT of 51 adult CD patients on medical therapy who were randomized to PEN with an unrestricted oral diet comprising 50% of daily caloric requirements (*n* = 26) or a full unrestricted diet (*n* = 25) [62]. After a mean follow-up of 11.9 months, relapse rates were significantly lower in the PEN group (34.6% vs. 64.0%; multivariate hazard ratio 0.40, 95% CI: 0.16–0.98). More recently, Yamamoto et al.’s prospective study of 40 CD patients compared a regimen of overnight continuous elemental diet infusion and a daytime low-fat diet (*n* = 20) with habitual diet (*n* = 20) [63]. ITT analysis found that patients consuming a habitual diet experienced higher rates of clinical relapse (65% vs. 25%, *p* = 0.03) and endoscopic inflammation scores (*p* = 0.04) at 1 year. Comparison of cytokine assays (IL-1beta, IL-6, TNF-alpha) on mucosal biopsies at baseline and at 1 year demonstrated increasing concentrations in the habitual diet group over time, and no significant change in the PEN group. Studies that have not demonstrated efficacy of PEN in maintaining sustained remission provided formula for 20–30% of caloric requirements, suggesting that efficacy is contingent upon formula providing >35% of total caloric requirements [64].

Evidence for the use of PEN in managing active UC is limited and evolving. While it may provide a dietary option, particularly for patients unable to tolerate a regular diet, its efficacy data are lacking, and it is not known whether its effects vary based on disease severity and host variables.

### 3.2. Effects on the Gut Microbiome

Little is known about PEN’s mechanisms of action, though it is felt to have overlapping mechanisms with EEN. In a prospective study of 41 pediatric patients with quiescent or mild CD assessing the effects of PEN with a formula providing ~25% of daily caloric requirements, relapse rates at 12 months were similarly low in both PEN (*n* = 22) and habitual diet (*n* = 19) groups [44]. PEN was associated with a small but significant reduction in alpha diversity and no changes in beta diversity. PEN was also associated with upregulation of phosphatidylcholines, however this was felt to reflect the lipid composition of the formula. In a subsequent untargeted metabolomic analysis in 34 participants from this cohort, Marques et al. observed different metabolomic signatures between PEN (*n* = 16) and habitual diet (*n* = 18) groups at 12 months [65]. Key identifiable metabolites responsible for this separation comprised dietary constituents, environmental contaminants, and products of human or bacterial metabolism, including [6]-gingerdiol 5-O-beta-D-glucopyranoside, alpha-allokainic acid, guaiazulene, tubulosine, L-olivosyl oleandolide, 2,4-dinitrophenol, three peptides (Glu-Ile, His-Met-Leu, and Val-Tyr-Ile), phosphatidyl serine 16.0/19.0, 1,3-dichlorobenzene, methyl 1-(propylsulfinyl)propyl disulfide, amino (methoxysulfinyl) pentasulfideand meta-tyrosine. The low disease activity in the study population, however, may have reduced the ability to detect metabolic responses that are more clearly linked to inflammatory activity. Further investigation into PEN’s effects on the gut microbiome and metabolome is warranted.

### 3.3. Summary

Studies to date have not consistently demonstrated the efficacy of PEN for the induction of remission in CD. In contrast, PEN may be an option for maintenance of remission, although its therapeutic potential likely depends on restriction of the oral diet in terms of both nutritional composition and caloric requirements. At the same time, a highly restricted oral diet may compromise long-term compliance, particularly in the setting of quiescent disease, and so less restrictive diets may be more feasible for maintenance therapy [58]. Standardization of PEN’s dietary criteria and methodology is necessary to better understand PEN’s role in CD management.

## 4. Crohn’s Disease Exclusion Diet

Based on insights from studying the effects of EEN and PEN, much effort was made to develop whole food diets that emphasize anti-inflammatory foods and exclude pro-inflammatory foods. The Crohn’s disease exclusion diet (CDED) was designed to be an anti-inflammatory whole foods diet that is prescribed with tapering regimens of PEN. It excludes dietary components hypothesized to negatively impact the gut microbiome, intestinal permeability and mucosal immune system [56]. As such, the diet emphasizes fresh whole foods and consumption of up to 18–20 g of fibre daily, and excludes gluten, baked goods, dairy products, certain animal fats, processed foods, food additives and emulsifiers [66].

The CDED has been studied using a phased approach during which there is gradual dietary expansion. The first six-week period is an induction phase and the most restrictive, with 50% of caloric intake supplied by PEN, usually with a polymeric formula [66,67]. The following six-week period is a step-down phase that reduces PEN to 25% of total energy requirements and re-introduces some grains, certain fish, red meat, nuts, legumes, and most fruits and vegetables [66,67,68]. More recently, a maintenance phase has been studied during which additional foods are slowly re-introduced every 6 weeks, which is meant to last at least 9 months [34,69].

### 4.1. Effects on Disease Activity

The CDED has emerged as a promising dietary strategy for CD and has been associated with improvements in clinical and biochemical outcomes in several studies. It was initially studied in a prospective study and case series in children and adults with active CD [66,70]. These studies demonstrated significant reductions in PCDAI and HBI scores and remission rates as high as 70%, with baseline disease severity and distribution emerging as predictors of response. However, the small sample sizes and uncontrolled study designs limit definitive conclusions regarding efficacy. The same group subsequently assessed the CDED in a RCT with 74 pediatric patients with mild-to-moderate CD over 12 weeks [24]. The control group received EEN for 6 weeks, followed by 25% PEN with an unrestricted diet for 6 weeks. ITT analysis revealed substantial and comparable rates of reduction in PCDAI scores, CRP and FCP between both groups at week 6 (median FCP in μg/g at baseline vs. week 6: CDED: 3126 vs. 1744, *p* = 0.002; EEN 2647 vs. 1021, *p* = 0.011; median delta FCP: CDED: −1473 vs. EEN: −948, *p* = 0.83). At week 12, the CDED group demonstrated significantly higher rates of sustained clinical and biochemical remission, while the EEN group saw a nonsignificant increase in FCP. The re-introduction of a free oral diet in the control arm may explain these observations. Similar findings were observed in more recent prospective studies that reported significant reductions in FCP over 12 weeks in both children and adults with CD [69,71].

In another study, Yanai et al. assessed the contribution of PEN to clinical outcomes in patients following the CDED [72]. In an open-label pilot RCT, biologic-naïve adults with mild-to-moderate CD were randomized to CDED + PEN (*n* = 19) or CDED alone (*n* = 21) for 24 weeks. There was a significant decrease in FCP observed at week 12 in both groups (median FCP [IQR] in μg/g—baseline: 262 [73–1092] vs. week 12: 97 [54–212], *p* = 0.0123). In fact, 40% of participants had an FCP below 100 μg/g at week 12. The study also demonstrated endoscopic improvement in both arms. Of 40 participants who underwent endoscopic evaluation at week 24, 14 (35%) demonstrated endoscopic remission, with similar rates in both groups (CDED + PEN: 8, CDED alone: 6, *p* = 0.70). There was a reduction of 5 points in median SES-CD score [IQR −6.2 to −1.0] in the subgroup of 22 participants with paired colonoscopies at baseline and week 24, representing a 72.8% decrease from baseline (*p* = 0.0025). These findings suggest that the CED may help maintain remission in some patients over the medium term, however confirmation in larger studies with longer follow-up is needed. It also suggests that the mechanisms underlying the beneficial effects of the CDED are likely driven by the exclusion of certain foods rather than the inclusion of PEN.

### 4.2. Effects on the Gut Microbiome

In Levine et al.’s RCT comparing CDED induction and step-down phases to EEN, clinical responses to both diets were associated with microbial compositional changes, namely shifts in bacterial diversity, an increase in the Firmicutes phylum, and a decrease in the Proteobacteria phylum, although changes at the genus level within Firmicutes varied by diet [24,34]. Fecal microbial analysis of diet responders at week 6 demonstrated significant reductions in *Haemophilus*, *Veillonella*, *Bifidobacterium*, *Prevotella*, *Anaerostipes* and Proteobacteria, specifically Gammaproteobacteria, and increases in *Oscillibacter*, *Anaerotruncus*, *Clostridiales* and butyrate-producing *Roseburia* [24]. Despite significant shifts in many of the same taxa in the EEN group, patterns of taxonomic changes differed between the groups. A stark deviation was observed between weeks 6 and 12 during which CDED responders demonstrated sustained compositional change while EEN responders demonstrated a return to pre-treatment microbial composition. This was particularly evident in Proteobacteria which has been reported to be increased in CD in some studies.

CDED non-responders exhibited increases in Lachnospiraceae, Erysipelotrichaceae, Actinomyces and Halomonas and a decrease in Bifidobacterium [24]. Non-responders of either diet at week 6 demonstrated less compositional change overall, which was in part due to narrower taxonomic shifts among Proteobacteria (more Gammaproteobacteria).

Taken together, these observations suggest that excluding certain dietary components is crucial for maintaining remission and the associated microbial changes, while an unrestricted diet may reverse these changes [24]. They also suggest that Proteobacteria may be involved in diet-associated microbial shifts in CD, although the causal role of these changes remain unclear.

### 4.3. Summary

A systematic review by Zhu et al. comprising seven studies concluded that CDED had similar efficacy to EEN in achieving clinical and biochemical remission, however it was superior to EEN in terms of tolerability and adherence [68]. The studies were heterogeneous in methodology, small in sample size, had limited long-term follow-up, and most lacked a control group. While larger, high-quality RCTs are needed to validate these findings and further explore the benefits of CDED with and without PEN, current evidence suggests that CDED may have potential as a long-term dietary strategy for inducing and maintaining remission in adults and children with mild-to-moderate CD. There is no evidence to date to support its use in UC.

## 5. Crohn’s Disease Treatment-with-Eating Diet

The Crohn’s disease treatment-with-eating diet (CD-TREAT) is a personalized anti-inflammatory solid food diet that seeks to replicate the composition and efficacy of EEN in CD while simultaneously increasing diet tolerability [25]. By excluding gluten, lactose, and alcohol and by emphasizing proteins, vitamins, minerals and fibre, it closely resembles the composition of EEN (MODULEN™ IBD, Nestlé, Vevey, Switzerland) and has been demonstrated to induce similar shifts in the gut microbiome and metabolome, intestinal inflammation, and clinical response.

### 5.1. Effects on Disease Activity

Svolos et al. investigated CD-TREAT in three parallel studies of healthy adults, an animal model, and an open-label pilot trial for pediatric CD [25]. In a crossover randomized controlled trial, 25 healthy adults completed a course of EEN (MODULEN™ IBD) and CD-TREAT for 7 days each, separated by a 14-day washout period. Participants found CD-TREAT to be more tolerable and easier to follow than EEN. Although gastrointestinal symptoms were uncommon to both diets, CD-TREAT had lower rates of abdominal pain and diarrhea (*p* = 0.005 for both). In the small open-label pilot trial, five children with active CD were given CD-TREAT over 8 weeks. Four participants (80%) demonstrated clinical response (defined as a weighted-PCDAI [wPCDAI] score decrease of ≥17.5), and three participants (60%) achieved clinical remission (defined as a wPCDAI score < 12.5). It also induced a biochemical response, with a significant reduction in FCP observed (mean decrease 918 ± 555 mg/kg, *p* = 0.002). However, the small sample size and uncontrolled design limit conclusions regarding clinical efficacy.

### 5.2. Effects on the Gut Microbiome

Both diets induced similar changes in the gut microbiome in healthy adults. While neither diet induced a significant change in alpha diversity, both interventions led to substantial shifts in beta diversity in the same direction [25]. EEN led to significant changes in the relative abundance of 58 (49.3%) genera and CD-TREAT led to significant changes in 38 (32.3%) genera. Similar directional shifts were observed in 28 of these genera for both diets, translating to 48.3% of EEN’s effect being reproduced by CD-TREAT and 73.7% of CD-TREAT’s effect being reproduced by EEN.

Similarly, there were overlapping metabolomic shifts observed between CD-TREAT and EEN in healthy adult volunteers. Both diets increased fecal pH to alkaline levels (mean pH increase: EEN: 1.3 ± 0.5 units, CD-TREAT: 0.9 ± 0.6 units, *p* < 0.001 for both) [25]. Fecal concentration of total sulfide increased with both diets (EEN 133.0 ± 80.5 nmol/g, CD-TREAT 54.3 ± 47.0 nmol/g, *p* < 0.001 for both), however, a decrease in free sulfide was observed after EEN only. Both diets led to significant reductions in mean concentrations of short-chain fatty acids (SCFAs) with a similar effect size (in μmol/g: acetate: EEN −27.4 ± 22.6, CD-TREAT −21.6 ± 20.4; propionate: EEN −5.7 ± 7.8, CD-TREAT −5.2 ± 7.9; butyrate: EEN −7.0 ± 7.4, CD-TREAT −10.2 ± 8.5). Branched-chain fatty acids (BCFAs) increased after EEN only, while valerate decreased after CD-TREAT only. Comparable findings were observed in a rat model, with similar shifts in microbiome, SCFAs, and histopathologic severity of ileitis. Microbial and metabolomic analyses were not performed in CD patients.

### 5.3. Summary

Although CD-TREAT is in its nascent stage of evaluation, preliminary findings suggest that it may offer a more tolerable food-based alternative to EEN with similar short-term clinical, biochemical, microbial, and metabolomic changes. However, the current evidence is limited, with clinical data derived primarily from a small pilot study involving only five pediatric patients. As such, CD-TREAT cannot yet be considered sufficiently validated for broad clinical implementation, and larger clinical trials are needed to confirm its efficacy and further characterize its microbial and metabolomic effects in active CD. Additionally, CD-TREAT includes ordinary foods that can be processed, which may raise concerns over the diet’s overall quality and long-term effects.

## 6. Tasty & Healthy Dietary Approach

The Tasty & Healthy (T&H) dietary approach is a guiding framework that encourages dietary diversity and minimizes rigidity. Initially developed for a variety of gastrointestinal disorders, T&H has been studied primarily in children and young adults with CD. It excludes pro-inflammatory dietary components, including processed foods, gluten, red meat, and dairy, with the exception of plain yogurt, and otherwise does not require specific ingredients or liquid formulas [26,73]. In this way, it is unique among the other diets discussed in this article in that it does not mandate a rigid structure, a feature that has been hypothesized to increase long-term compliance.

Its tolerability and efficacy were studied in a RCT, in which 83 children and young adults with mild-to-moderate CD were randomized to T&H (*n* = 41) or EEN (*n* = 42) for 8 weeks [26]. There was a significant reduction in FCP in both arms, and no difference between groups at week 8 (FCP < 250 at week 8: T&H 38% vs. EEN 41%, *p* = 0.84). There were also significant reductions in erythrocyte sedimentation rate (ESR) and CRP, with no differences in these parameters between groups at week 8. ITT analysis found higher rates of mucosal healing in the T&H group, as assessed by the Mucosal Inflammation Noninvasive (MINI) index (MINI < 8 at week 8: T&H 54% vs. EEN 41%, *p* = 0.026). Further, T&H was substantially better tolerated than EEN (T&H 88% vs. EEN 52%, OR 6.3, 95% CI 2.2–22, *p* < 0.001).

Although there are limited data evaluating its use in IBD, the T&H approach has shown comparable efficacy to EEN with better tolerability, and offers an emerging alternative to rigid, structured diets. Further studies are needed to validate these findings and assess long-term outcomes.

## 7. Specific Carbohydrate Diet

The specific carbohydrate diet (SCD) was first introduced in the 1920s by Dr. Sidney Haas as a treatment for celiac disease [45,74]. It was later popularized as a treatment for IBD after Elaine Gottschall’s book *Breaking the Vicious Cycle: Intestinal Health through Diet* was published in 1987, which described the use of SCD in managing various gastrointestinal disorders including IBD [75]. The premise of SCD is that complex carbohydrates exert a strong influence on the intestinal microbiome, mucosal integrity and immune function [76]. This is based on the notion that disaccharides and polysaccharides are poorly absorbed, which leads to bacterial overgrowth and excessive fermentation of undigested carbohydrates, in turn resulting in the production of organic acids, such as lactic acid and acetic acid, that can precipitate intestinal inflammation [76,77]. As such, the diet is preferential towards monosaccharides while excluding disaccharides and most polysaccharides. SCD consists of three phases: (1) a restrictive introductory diet for up to one week, consisting of dry curd cottage cheese, homemade yogurt, eggs, apple cider, chicken soup, broiled fish, and beef patties; (2) the gradual introduction of cooked fruits and vegetables; and (3) a maintenance phase, with gradual dietary expansion to include raw fruits and vegetables, plant-based proteins, poultry and meats. Gotschall also advocated for further restrictions to extend to juices from concentrate, certain prepared foods, supplements, and non-essential medications since they could contain added illegal sugars that are not included in the label ingredients, unless stated otherwise by the manufacturers [77]. SCD’s efficacy should be determined after at least one month, however Gottschall recommended strict compliance for at least one year after achieving clinical remission for best results [77].

The restrictive framework of SCD, in terms of both its nutritional composition and rigid adherence, has the potential to cause nutritional deficiencies. Its limited dairy products and exclusion of fortified grains increase the risk of deficiencies in folate, thiamine, other B-vitamins, calcium, and vitamin D. A 12-week prospective study in pediatric IBD patients following SCD found that underconsumption of vitamins B1, B9, and D; calcium; phosphorus; magnesium; and zinc below the recommended daily allowance was common [78].

### 7.1. Effects on Disease Activity

The majority of studies evaluating the efficacy of SCD are retrospective. In one prospective study of nine pediatric patients with active CD, Cohen et al. demonstrated substantial clinical improvement after 12 weeks of SCD that was sustained to 1 year, reinforcing earlier studies that SCD provides durable long-term symptomatic benefit (HBI mean ± SE—baseline: 3.3 ± 2.0 vs. week 12: 0.6 ± 1.3 vs. week 52: 0.1 ± 0.4, *p* = 0.007 and *p* = 0.016 for weeks 12 and 52 compared to baseline, respectively; PCDAI mean ± SE—baseline: 21.1 ± 5.9 vs. week 12: 7.8 ± 7.1 vs. week 52: 5.4 ± 5.5, *p* = 0.011 and *p* = 0.027 for weeks 12 and 52 compared to baseline, respectively) [27]. Despite sustained symptomatic benefit, effects on mucosal healing were transient, as capsule endoscopy Lewis scores decreased significantly at 12 weeks but this improvement was not sustained at 1 year (Lewis score mean ± SE—baseline: 2153 ± 732 vs. week 12: 960 ± 433 vs. week 52: 1046 ± 372, *p* = 0.012 and *p* = 0.091 for weeks 12 and 52 compared to baseline, respectively). In a multicentre prospective study of 12 pediatric patients with CD (*n* = 9) and UC (*n* = 3), SCD led to symptomatic improvement, however there was no significant change in intestinal inflammation at 12 weeks (mean PCDAI—baseline 28.1 ± 8.8 vs. week 12: 4.6 ± 10.3; mean Pediatric Ulcerative Colitis Activity Index [PUCAI]—baseline: 28.3 ± 23.1 vs. week 12: 6.7 ± 11.6) [79]. Mean FCP decreased from 642.3 ± 648.6 μg/g at baseline to 202.6 ± 245.2 μg/g at week 12 at one recruitment site (*n* = 9), while it increased from 110.0 ± 100.0 μg/g at baseline to 209.0 ± 159.8 μg/g at week 12 at the other recruitment site (*n* = 3) (*p* = 0.10, *p* = 0.67 respectively).

More recently, Lewis et al. conducted the DINE-CD trial, a RCT comparing the clinical and biochemical effects of SCD and Mediterranean diet (MD) in 191 adults with mild-to-moderate CD [80]. This was a superiority study that hypothesized that SCD is superior in achieving the study endpoints. The trial was 12 weeks in duration, with the primary end point being symptomatic remission (defined as sCDAI < 150) at week 6 and a key secondary end point being response in FCP (defined as reduction of FCP to < 250 μg/g and by >50% from baseline among those with baseline FCP > 250 μg/g) at week 6. Participants received prepared foods according to their assigned diets for the first 6 weeks, and independently followed their assigned diets for the remaining 6 weeks. The primary endpoint was not achieved, as rates of symptomatic remission were similar among both groups (SCD 46.5%, MD 43.5%, *p* = 0.77). Similarly, both diets exerted comparable effects on intestinal inflammation. Among patients with elevated FCP at baseline (*n* = 36), biochemical response rates at week 6 were 34.8% in the SCD arm and 30.8% in the MD arm (*p* = 0.83). Among those with symptomatic remission, combined symptomatic remission and FCP response was uncommon (SCD 26.1%, MD 7.7%, *p* = 0.25). Due to the lack of a control arm, no conclusions can be drawn regarding a possible therapeutic advantage of these diets over the patients’ habitual diets. The authors concluded that MD may be preferrable over SCD in mild-to-moderate CD due to its general health benefits and the restrictive framework of SCD, which may limit long-term adherence and increase the risk of nutritional deficiencies.

### 7.2. Effects on the Gut Microbiome

Adherence to SCD has been associated with increased gut microbial diversity in IBD patients. It has been proposed that SCD may be associated with shifts in microbial composition, including changes in taxa linked to inflammatory activity, which may in turn relate to changes in intestinal inflammation. In Suskind et al.’s prospective study in pediatric CD and UC patients, there was a moderate increase in alpha diversity, with median Shannon diversity increasing from 2.48 at baseline to 2.65 at week 12 [79]. There was a decrease in proteobacteria across the cohort. Suskind et al. subsequently conducted a multi-omics pilot RCT comparing three varieties of SCD in 18 pediatric patients with mild-to-moderate CD over 12 weeks, though only 10 patients completed the study [45]. The diets were SCD, modified SCD (SCD plus oats and rice), and a whole foods diet (elimination of wheat, corn, sugar, milk and food additives), and all meals were catered. All 10 patients demonstrated clinical remission at 12 weeks with no differences between dietary assignments. The modified SCD group demonstrated the greatest reduction in FCP (baseline: 697 ± 520 mg/kg vs. week 12: 157 ± 156 mg/kg). Stool was collected from five patients for further analysis. Shifts in microbial composition were observed in all five patients and were largely patient-specific, however there was a broader trend of increasing diversity, with Inverse Simpson indices ranging from 4.67–14.09 at baseline compared to 10.93–17.76 at week 12. There was an increase in abundance of *Blautia*, *Lachnospiraceae*, *Faecalibacterium*, *Roseburia*, *Anaerobutyricum hallii* and *Eubacterium eligens*, and a decrease in abundance of *Escherichia coli* and a strain of *Faecalibacterium prausnitzii*. Metaproteomic analysis revealed decreased metabolic activity related to starches and simple sugars in the SCD group and an increase in some metabolic functions related to starch and sucrose metabolism in the modified SCD group, possibly reflecting the difference in carbohydrate content between the two diets. It also demonstrated increased amino acid metabolism across all groups. Patients assigned to the whole foods diet tended to have decreases in glutamate synthase, methionine synthase and 2-dehydro-3-deoxy-D-pentonate aldolase. The shifts in microbial composition and carbohydrate and protein metabolism likely occurred in response to dietary changes, particularly reduced carbohydrate content, leading to functional changes in the microbiome in sourcing energy from more complex nutrients and potentially modulating intestinal inflammation. However, larger studies with control arms are needed to verify the significance of these results.

### 7.3. Summary

There is a paucity of high-quality studies assessing the efficacy of SCD in IBD. The existing data suggest that SCD can improve symptoms but it does not appear to have a durable effect on intestinal inflammation. It is also associated with micronutrient deficiencies, and the strict protocol may pave the way for disordered eating such as orthorexia nervosa [77]. As a result, the current literature does not support the use of SCD in the management of IBD.

## 8. IBD-Anti-Inflammatory Diet

The IBD-Anti-Inflammatory Diet (IBD-AID) is derived from SCD and further modified based on the current understanding of the gut microbiome [77,81]. It leverages foods rich in omega-3 fatty acids and pre- and probiotics in order to restore homeostasis between beneficial and pathogenic taxa. It therefore incorporates foods that are excluded (“forbidden” or “illegal”) in SCD [81]. The diet consists of five core principles: (1) limitation of certain carbohydrates such as refined sugars, gluten-based grains, and certain starches felt to stimulate pathogenic, inflammation-promoting taxa; (2) consumption of food-based pre- and probiotics to cultivate a healthy microbiome; (3) a reduction in saturated and trans fats and an increase in unsaturated fats in order to attenuate inflammation; (4) provide a framework for a varied and well-balanced diet that addresses both nutritional deficiencies and food intolerances; and (5) a phased approach to introducing foods and textures based on clinical status in order to optimize nutrient absorption and alleviate symptoms [77,81].

### 8.1. Effects on Disease Activity

A small case series of 11 adult patients (8 CD, 3 UC) found that compliance with the diet for at least 4 weeks was associated with symptomatic remission and de-escalation of medical therapy (mean HBI [range]—baseline: 11 [1–20] vs. follow-up: 1.5 [0–3]; mean Modified Truelove and Witts Severity Index [range]—baseline: 7 [6–8] vs. follow-up: 0) [81].

### 8.2. Effects on the Gut Microbiome

The diet’s effect on the gut microbiome was studied in one prospective single-arm pre-post intervention pilot trial involving 19 patients (12 CD, 7 UC), who kept their habitual diets for 6 weeks followed by IBD-AID for 8 weeks [46]. Stool was collected on a biweekly basis throughout the study. Among the top 10 taxa that increased in abundance in both CD and UC patients during the intervention were SCFA-producing bacteria that belonged predominantly to the Clostridia and Bacteroidia classes, including *Roseburia hominis*, *Eubacterium eligens*, *Faecalibacterium prausnitzii*, *Blautia obeum*, *Bacteroides dorei*, and *Bacteroides vulgatus*. A number of these taxa are commonly depleted in IBD patients. In contrast, there was decreased abundance of taxa belonging to Bacteroidia, Coriobacteriia, and Clostridia classes. Prebiotics, probiotics and beneficial foods of IBD-AID shared a positive correlation with favourable taxa enriched during the intervention, and a negative correlation with taxa enriched at baseline. Opposite correlations were observed with adverse foods of IBD-AID.

### 8.3. Summary

IBD-AID is a potential therapeutic avenue for IBD, with preliminary data demonstrating clinical efficacy and beneficial taxonomic shifts. Large RCTs evaluating its effects on intestinal inflammation and microbial composition and function would be valuable for assessing its therapeutic potential and further understanding its mechanisms of action.

## 9. Autoimmune Protocol

The autoimmune protocol (AIP) is an extension of the Paleolithic diet that incorporates anti-inflammatory dietary principles previously studied in IBD in order to reduce inflammation, dysbiosis, and symptoms [28]. It consists of an initial phase eliminating refined sugars, food additives, grains, legumes, nightshades, nuts, seeds and seed oils, dairy, eggs, coffee, and alcohol. It also encourages fresh nutrient-rich foods, bone broth, and fermented foods. The elimination phase is followed by a maintenance phase that is intended to last until there is clinical improvement. Food groups are then gradually re-introduced, enabling patients to identify dietary triggers of symptoms as they gradually broaden their diet. A prospective study of 15 adult patients with active IBD (9 CD, 6 UC) evaluated the efficacy of AIP in optimizing disease activity over a 6-week elimination phase, followed by a 5-week maintenance phase. There were significant improvements in HBI and partial Mayo scores (PMS) at week 6, which were maintained until week 11 (mean HBI [SD]—baseline: 6.7 [1.5], week 6: 3.3 [1.8], week 11: 3.4 [2.6], *p* = 0.001 and *p* = 0.004 for weeks 6 and 11 compared to baseline, respectively; mean PMS [SD]—baseline: 5.8 [1.2], week 6: 1.2 [2.0], week 12: 1.0 [2.0], *p* = 0.01 and *p* = 0.007 for weeks 6 and 11 compared to baseline, respectively). There was no significant difference in rates of clinical remission between CD and UC patients. There was a decrease in mean FCP from 471 (SD 562) at baseline to 112 (SD 104) at week 11, however this did not reach significance. Seven patients underwent endoscopic evaluation at week 11, with the majority demonstrating improvement in the simple endoscopic score for CD (*n* = 1), Rutgeerts score (*n* = 1), and Mayo endoscopy subscore (*n* = 4). Given the small sample size, lack of randomized trial design, and absence of a control group, the efficacy of AIP in improving disease activity is unclear, and larger, randomized controlled studies are warranted.

## 10. Low-Sulfur Diet

There is substantial evidence that excessive hydrogen sulfide (H_2_S) and nitric oxide (NO) contribute to the development of IBD, particularly UC. Increased H_2_S and NO have been associated with several destructive processes including impairment of mitochondrial function, reduction in butyrate uptake by colonocytes, and increased production of reactive oxygen species [82,83,84]. These mechanisms contribute to loss of epithelial barrier function and subsequent inflammation and have been implicated in UC pathogenesis [47].

Increased concentrations of H_2_S and sulfate-reducing bacteria (SRB) have been detected in patients with UC, with a positive correlation between sulfide production and disease activity [85]. This is likely due to impairment of thiosulfate sulfurtransferase enzymes in the colonic mucosa that metabolize and detoxify H_2_S, and increased expression or activity of SRB stimulated by sulfated amino acids. In fact, a positive correlation between thiosulfate sulfurtransferase impairment and disease activity has been observed. Further, SRB was found to be the predominant bacterial cluster in UC patients compared to the minority cluster in healthy controls.

Dietary modulation of sulfur content has been proposed as a means of reducing H_2_S and studied to a limited degree [86,87]. This notion is supported by the association observed between increased dietary sulfur and sulfate with UC relapse [88]. Dietary sulfur content, however, is not well-described given its ubiquity in the food supply and absence as a listed nutrient in major nutritional databases, including the USDA Food Composition Database [89]. Therefore, an accepted method of estimating dietary sulfur content is by quantifying consumption of sulfated amino acids. Sulfated amino acids, such as cysteine and methionine, are the primary dietary sources of sulfur and are found predominantly in proteins, particularly animal proteins. This approach does not encompass sulfur-containing food additives or modifiers including sulfiting agents, sulfuric acid, and sulfated polysaccharides, such as carrageenan [89]. Further, mechanistic data using in vitro gas-profiling technology found that readily fermentable fibres suppressed H_2_S production more effectively than poorly fermentable fibres and 5-aminosalicylate acid [90]. On this basis, a low-sulfur diet can be characterized as a plant-based, protein-restricted diet devoid of certain food additives and enriched with fermentable fibre.

### 10.1. Effects on Disease Activity

A handful of studies explored low-sulfur dietary interventions in UC, which predominantly fell within a plant-based, semi-vegetarian dietary framework [89]. Although these tended to demonstrate clinical benefit, much of these data are restricted to small case series or case reports [83,89,91]. More recently, the 4-Strategies-to-Sulfide-Reduction (4-SURE) diet was assessed in an 8-week open-label feasibility study in 28 adult patients with mild-to-moderate active UC, most of whom had left-sided or pancolonic disease [47]. The parameters of the diet included (1) 10–15 g/day of resistant starch and 5 g/day of a slowly fermentable non-starch polysaccharide; (2) restricted total protein intake from animal and plant sources up to 75–90 g/day (≤1.2 g/kg/day); (3) sulfated amino acid consumption limited to ≤1.5–2.0 g/day; and (4) avoidance of sulfite/sulfate, nitrite/nitrate, and carrageenan food additives. In ITT analysis, clinical response was achieved in 46% of participants, defined as a reduction in PMS by ≥2 points. Endoscopic response, defined as a reduction in Mayo endoscopic subscore by ≥1 point or reduction in Ulcerative Colitis Endoscopic Index of Severity (UCEIS) by ≥2 points, was observed in 36% of participants (*p* = 0.01 for UCEIS). Median FCP concentration decreased by 131 μg/g (*p* = 0.02). The per-protocol analysis (*n =* 24) found that 33% of participants had a substantial improvement in total Mayo score (median [IQR]—baseline: 6 [5–8] vs. week 8: 4 [3–6], *p* < 0.0001). The diet was well-tolerated throughout the duration of the study.

Soon after, Strauss et al. conducted an open-label RCT assessing the effects of modulating sulfur intake within a Mediterranean-like dietary pattern (*n* = 22) compared with a habitual diet (*n* = 18) over 8 weeks in adults with UC [48]. The participants were heterogeneous in terms of baseline disease activity, although the majority had active disease per PMS (baseline median PMS = 3 [IQR 1–5]). There was a small reduction in PMS (median 2 points) observed in both groups (median [IQR] PMS: low-sulfur diet—baseline: 2 [0–5] vs. week 8: 0 [0 = 1], *p* = 0.003; habitual diet—baseline: 3.5 [1–5] vs. week 8: 1 [0–1.8], *p* = 0.007). However, there were no significant intra- or inter-group changes in FCP; in fact, both groups demonstrated an increase in FCP (median FCP [IQR] mcg/g: low-sulfur diet—baseline: 73 [30–989] vs. week 8: 172 [37–844], *p* = 0.72; habitual diet—baseline: 235 [73–3945] vs. week 8: 271 [73–2435], *p* = 0.68). Notably, UC flares were managed with conventional medical care including escalation of medical therapy which may have blunted the dietary response.

### 10.2. Effects on the Gut Microbiome

In the 4-SURE study, there was a 69% increase in total daily excretion of SCFA (*p* = 0.0001), with similar increases in butyrate, acetate, and propionate (*p* ≤ 0.001) [47]. While reductions in total BCFA and ammonia concentrations and daily excretions occurred, they did not reach significance. The BCFA:SCFA ratio decreased by 27% (mean difference −1.34; 95% CI: −2.28%, −0.40%; *p* = 0.007). These findings indicate a preference for carbohydrate fermentation over protein fermentation.

Strauss et al. found a substantially higher concentration of valeric acid in the low-sulfur diet arm at week 8 (median [IQR]—low-sulfur diet: 704 [441–958] vs. habitual diet: 360 [58–677], *p* = 0.05) [48]. There were no significant differences in the concentrations of the other SCFAs within and between groups. Further, there was a marked difference in glycochenodeoxycholic acid, a conjugated bile acid, between the groups, both at baseline and at week 8 (median [IQR]: baseline—low-sulfur diet: 0.1 vs. habitual diet: 0.4, *p* = 0.005; week 8—low-sulfur diet: 0.2 vs. habitual diet: 0.4, *p* = 0.02), though no differences for the rest of the bile acids. Perturbations in bile acid metabolism have been associated with inflammation and IBD, with a correlation between conjugated bile acids and disease activity. These findings were reported alongside lower PMS scores in the intervention group.

In our work in Primary Sclerosing Cholangitis (PSC), a chronic inflammatory cholestatic liver disease closely associated with IBD and UC in particular, we conducted a RCT comparing the effects of the low-sulfur diet (*n* = 10) and SCD (*n* = 10) over 8 weeks in 20 patients with PSC, 13 of whom had concomitant IBD (3 CD, 10 UC) [92,93]. The low-sulfur diet induced significant shifts in SRB, such as *Eggerthella lenta*. This led to significant alterations in concentrations of sulfur-containing metabolites, including the depletion of methionine and methionine sulfone and an increase in taurine. It also induced significant changes in luminal bile acids, including an elevation in cholic acid and a decrease in taurocholic acid. These observations support the notion that the low-sulfur diet exerts a strong influence over sulfur metabolism and H_2_S production.

### 10.3. Summary

While the low-sulfur diet appears effective in reducing luminal symptoms, the evidence for improving intestinal inflammation is inconsistent. The paucity of high-quality studies evaluating its effects on disease activity warrant further exploration in larger, randomized controlled studies with a standardized diet regimen.

## 11. Ulcerative Colitis Exclusion Diet

The UC Exclusion Diet (UCED) was developed with a focus on dietary components that may influence parameters associated with UC, namely goblet cells, mucous permeability, and microbial composition [94]. It emphasizes tryptophan and natural sources of pectin and resistant starch while restricting sulfated amino acids and total protein, heme, animal fats, saturated and polyunsaturated fats, and food additives. Its framework involves mandatory foods, primarily certain fruits and vegetables; controlled amounts of chicken, eggs, dairy products, and grain products, among other foods; and elimination of certain foods, such as red meat and processed foods [94,95]. It consists of a 6-week elimination phase followed by a 6-week expansion phase.

### 11.1. Effects on Disease Activity

UCED was first studied in a 12-week open-label pilot study in 24 children with active UC on stable maintenance therapy [95]. The primary outcome was clinical remission at week 6 with UCED as the sole intervention. ITT analysis demonstrated a response rate of 70.8% and remission rate of 37.5% at week 6. Median [IQR] PUCAI decreased from 35 [30–40] at baseline to 12.5 [5–30] at week 6 (*p* = 0.001). Median [IQR] FCP decreased from 818 [630.0–1880.0] μg/g at baseline to 592.0 [140.7–1555.0] μg/g at week 6 (*p* > 0.05). Among the eight participants who received antibiotic rescue therapy, four (50%) achieved remission, although inflammatory markers did not improve.

UCED was subsequently studied in the CRAFT UC blinded RCT in 51 adults with active UC [94]. There were three study arms: Group 1—free diet for patients and single donor standard fecal microbiota transplantation (FMT) without dietary conditioning of the donor (*n* = 17); Group 2—UCED for patients and FMT as above with dietary pre-conditioning of the donor for 14 days (*n* = 19); and Group 3—UCED alone for patients (*n* = 15). Mucosal healing (defined as Mayo score 0) was only observed in Group 3, with 3/15 (20%) achieving this endpoint compared to none in Groups 1 and 2 (*p* = 0.022). Post-FMT stool analysis from Groups 1 and 2 found beneficial microbial compositional and functional changes in Group 2, including increased alpha diversity (*p* = 0.019), a shift towards the donors’ microbial composition (*p* = 0.008), and enrichment in eight metabolic pathways including those involved in branched chain amino acid synthesis and ribonucleotide synthesis, which were not observed in Group 1 [96]. However, it is unclear if these changes were driven by dietary conditioning of donors or the use of UCED post-FMT in patients. Stool analysis was not performed in Group 3, though such knowledge may provide insight into the UCED’s mechanism of action and potentially allow for further dietary customization.

### 11.2. Summary

UCED demonstrated a promising ability to reduce intestinal inflammation in active UC in one RCT, however the sample size was small due to premature closure of the study for futility. Its anti-inflammatory potential provides a basis for further exploration in larger clinical trials. Dietary modulation of the gut microbiome was an important consideration in the design of the diet, however its effects on microbial composition and function are not currently known.

## 12. Mediterranean Diet

The Mediterranean diet (MD) is characterized by high consumption of fruits, vegetables, plant-based proteins, whole grains, and unsaturated fats, with olive oil being the predominant source of fat; moderate consumption of fish, seafood, poultry, animal products, and wine; and reduced consumption of saturated fats, meat and processed foods [49,97]. It can beneficially modulate the gut microbiome and metabolome, particularly with increased abundance of fibre-degrading bacteria and production of SCFAs, which are theorized to reduce the risk of IBD [31,98,99]. It has also demonstrated potential to induce favourable immunomodulatory effects [100] and epigenetic processes related to inflammation [101]. It is believed that its advantageous effects stem both from its exclusion of deleterious dietary components and its inclusion of beneficial ones, including fibre; polyphenols that play an antioxidant role; and an anti-inflammatory fatty acid profile rich in monounsaturated fatty acids, moderate in saturated fatty acids, and low in *n*-6 polyunsaturated fatty acids (PUFAs) and some *n*-3 PUFAs [49,102]. It has been widely studied and associated with positive outcomes in several chronic diseases, including malignancies, cardiovascular disease [103,104], and immune-mediated disorders, such as psoriasis [105,106] and rheumatoid arthritis [107,108,109]. Epidemiologic studies have observed an inverse association between IBD development and adherence to MD [110,111,112]. Further, MD has been associated with reduced symptoms and improved quality of life in IBD patients [50,113].

### 12.1. Effects on Disease Activity

The DINE-CD trial (see SCD above) found that MD resulted in a FCP response rate of 30.8%, which is lower relative to other diets studied in active CD [80,102]. It should be noted, however, that only a minority of participants had elevated FCP at baseline. More recently, a RCT of 100 patients, aged 12–18 years with mild-to-moderate active IBD (54 CD, 46 UC) on stable medical therapy, compared the efficacy of MD versus their habitual diets over 12 weeks [50]. The majority of patients in both arms had achieved clinical remission at 12 weeks, however PCDAI and PUCAI scores were significantly lower in the MD group relative to the control group (mean ± SD: PCDAI: MD 6.4 ± 8.1 vs. habitual diet 10.8 ± 7.4, *p* = 0.02; PUCAI: MD 7.6 ± 11.2 vs. habitual diet 9.2 ± 7.5, *p* = 0.04). Similarly, both groups demonstrated improvement in intestinal inflammation; however, the difference was greater in the MD group (FCP mean ± SD µg/g—baseline: 622.4 ± 411.5 vs. habitual diet 645.8 ± 435.7, *p* = 0.88; week 12: MD 221.5 ± 88.5 vs. habitual diet 395.7 ± 194.4, *p* = 0.03). Serologic inflammatory markers (including CRP, TNF-α, IL-17, IL-12, and IL-13) also decreased in both groups, with greater reductions observed in the MD group. An Italian study comparing dietary intake of 125 pediatric patients with quiescent IBD to healthy controls found an inverse correlation between MD adherence and FCP. Specifically, patients with FCP < 70 mcg/g reported higher Mediterranean Diet Quality Index for Children and Adolescents (KIDMED) scores compared to patients with FCP ≥ 70 mcg/g (KIDMED score mean ± SEM: 5.82 ± 2.35 vs. 4.85 ± 2.16; *p* = 0.027) [112].

A Canadian RCT in 28 adults with quiescent UC compared the effects of MD (*n* = 15) and habitual diet (*n* = 13) on intestinal inflammation and gut microbiome over 12 weeks [49]. While the majority (71%) of participants had FCP < 100 μg/g at baseline, 75% participants in the control group had FCP > 100 μg/g at week 12 compared to 20% in the MD group. The majority (87%) of participants in the MD group had maintenance or reduction in FCP, with no significant difference overall in FCP between baseline and week 12. In contrast, there was a substantial increase in FCP over 12 weeks in the control group, with half of this group experiencing an increase in FCP of >50% (*p* = 0.0488).

In an Italian interventional study, 142 adult IBD patients (84 UC, 58 CD) followed MD for 6 months, and dietary effects on nutritional status, hepatic steatosis, IBD activity and quality of life were assessed [97]. At 6 months, there was a significant decrease in CRP and FCP in both UC and CD patients, which mirrored improvement in symptoms and quality of life. Among UC patients with complete data who maintained stable medical therapy (*n* = 56 for CRP, *n* = 57 for FCP), the proportion of patients with elevated CRP decreased from 50% at baseline to 37.5% at 6 months (*p* = 0.013), and those with FCP > 250 mg/kg decreased from 43.8% to 28.1% (*p* = 0.049). Similarly, among the CD patients (*n* = 49 for CRP, *n* = 40 for FCP), the proportion with elevated CRP decreased from 44.9% to 26.5% (*p* = 0.035), and those with FCP > 250 mg/kg decreased from 45% to 27.5% (*p* = 0.035). MD adherence was also associated with favourable metabolic outcomes including improvement in hepatic steatosis and reduction in BMI and waist circumference.

### 12.2. Effects on the Gut Microbiome

In Haskey et al.’s study in adults with quiescent UC, MD did not alter alpha diversity; however, it induced significant and beneficial shifts in beta diversity [49]. Positive taxonomic associations with MD included *Alistipes finegoldii* and *Flavonifractor plautii*, which have been shown to be protective in animal models of colitis, as well as *Clostridium boltae*, *Ruminococcus bromii*, *Blautia A* spp., *Flavonifractor plautii*, and *Lactococcus lactis*, which are involved in the metabolism of butyrate, catechins, additional polyphenols, and other food substrates that regulate host health. In contrast, the taxa most negatively associated with MD included opportunistic pathogens *Veillonella dispar*, *Veillonella tobetsuensis*, *Prevotella copri*, and *Streptococcus australis*. *Prevotella copri* is also associated with autoimmune diseases including CD. Further, MD was associated with significantly higher concentrations of anti-inflammatory SCFAs compared to the control group (total SCFAs *p* = 0.0129, butyric acid *p* = 0.0287, acetic acid *p* = 0.0325, valeric acid *p* = 0.0083).

A Canadian controlled-intervention study explored diet–microbiome interactions in 40 adult patients with quiescent CD over 12 weeks [114]. Individuals who reported low consumption of fibre and high consumption of red and processed meat at baseline (*n* = 15) were assigned to a non-diversified diet (NDD) and received a dietary intervention incorporating parameters of MD, while the remaining participants (*n* = 25) were assigned to a diversified diet (DD) control group and received conventional care. Baseline microbial composition varied between groups in terms of beta diversity (*p* = 0.003) and relative abundance (mean ± SD) of Proteobacteria (NDD 7.2 ± 11.9% vs. DD 1.8 ± 4.4%, *p* = 0.003), *Faecalibacterium* (NDD 3.3 ± 5.4% vs. DD 8.5 ± 10.6%, *p* = 0.03), and *Escherichia*/*Shigella* (NDD 6.9 ± 12.2% vs. DD 1.6 ± 4.4%, *p* = 0.03). At 12 weeks, there was no difference in beta diversity between the groups (*p* = 0.43). The MD-like intervention in the NDD group resulted in an increase in *Faecalibacterium* (*p* = 0.049) such that it resembled the DD group (*p* = 0.84). It also led to decreases in the relative abundance of *Proteobacteria* and *Escherichia*/*Shigella* in the NDD group such that they were lower compared to the DD group at week 12 (*p* = 0.03 and *p* = 0.02, respectively).

MD has also demonstrated potential to modulate inflammation and epigenetic mechanisms [115]. In a small pilot interventional trial, eight adult patients with active CD were prescribed a MD-inspired diet for 6 weeks [99]. Post-intervention transcriptomic analysis revealed significantly altered expression of more than 3500 genes including genes involved in regulating NF-κB activity, which is crucial to regulating immune response, and the JAK/STAT pathway, which is involved in IBD. While there were no significant taxonomic changes post-intervention, there was a trend towards normalization of taxa known to have altered expression in CD. Specifically, there was an increase in the abundance of Bacteroidetes (17.89% to 18.74%), *Clostridium* cluster IV (19.2% to 21.86%), and *Clostridium* cluster XIVa (26.78% to 28.79%) and a decrease in the abundance of Proteobacteria (5.93% to 5.48%) and Bacillaceae (4.65% to 4.21%).

### 12.3. Summary

MD exerts favourable mechanistic effects in IBD, such as increasing gut microbial diversity, cultivating growth of beneficial taxa, modulating microbial function, and attenuating inflammation. It is associated with favourable outcomes in both CD and UC; however, the clinical and biochemical improvement has been moderate overall, and there is no evidence thus far that MD is superior to other diets in IBD. MD may be considered a health-promoting dietary pattern rather than a specific therapeutic intervention for IBD. This view is supported by its broader health benefits, widespread availability, less restrictive framework compared to other diets for IBD, with generally modest effects on intestinal inflammation in IBD. It therefore remains of interest for further evaluation in prospective studies or RCTs. MD may also be advantageous in individuals at high risk for developing IBD when considering the epidemiological data demonstrating IBD risk reduction with increased MD adherence [1].

## 13. Comparative Synthesis of Dietary Interventions in IBD

There is substantial heterogeneity in the clinical efficacy, objective disease activity endpoints, and durability of response among dietary interventions and IBD. Interpretation of their efficacy is further limited by variability in study methodology, including study design, dietary protocols, and durations, study endpoints, sample size and dietary adherence [116]. Further, few studies incorporate baseline and/or post-intervention objective disease activity assessment, including by endoscopy. The strongest evidence has been in CD, whereas UC is substantially less represented. This imbalance limits definitive conclusions regarding dietary efficacy in UC and represents a key gap for future dietary intervention studies.

**Figure 2:**
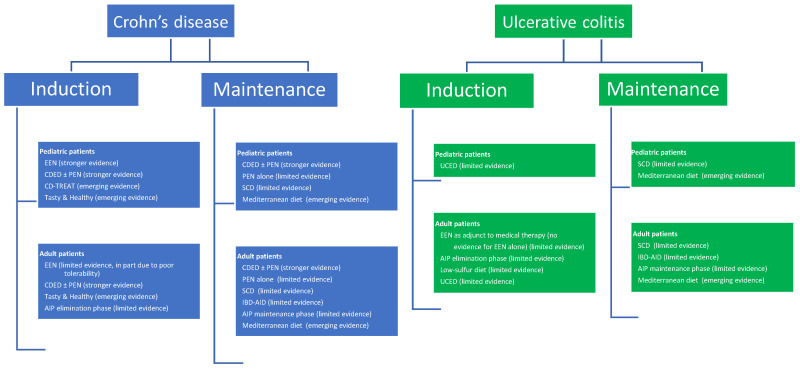
Decision-support algorithm for dietary interventions in IBD. This algorithm is intended as a general guide and should be tailored to patient-specific factors, including disease characteristics, nutritional needs, patient preferences, and the broader clinical context. EEN = exclusive enteral nutrition; PEN = partial enteral nutrition; CDED = Crohn’s disease exclusion diet; CD-TREAT = Crohn’s disease treatment-with-eating diet; SCD = specific carbohydrate diet; IBD-AID = IBD-Anti-Inflammatory Diet; AIP = autoimmune protocol; UCED = ulcerative colitis exclusion diet.

The most robust evidence is available for EEN, with RCTs and meta-analyses demonstrating approximately 80% pediatric remission rates, efficacy comparable to corticosteroids, and superior mucosal healing compared to steroids, although adult effectiveness is constrained by adherence (Figure 2). The CDED, particularly with PEN, shows comparable induction efficacy to EEN in pediatric cohorts and improved maintenance of remission, with better tolerability and sustained biochemical response. PEN alone appears more relevant for maintenance than induction, with reduced relapse risk but inconsistent effects on inflammatory biomarkers, likely reflecting heterogeneity in dietary composition and adherence. CD-TREAT reproduces EEN-like microbial and metabolomic signatures with early and sustained biochemical response but remains limited by a small study and lack of long-term or mucosal endpoints. Similarly, despite comparable short-term biochemical and clinical outcomes to EEN and improved tolerability, the evidence for the T&H diet is restricted to a single controlled study without long-term outcomes, which limits insight into its durability and generalizability. MD demonstrates modest but consistent reductions in inflammatory markers and improved clinical indices in both CD and UC, suggesting that it may be well-positioned as a sustainable adjunct rather than induction therapy. In contrast, SCD, IBD-AID, AIP, low-sulfur diet and UCED are supported mainly by small or uncontrolled studies, with frequent symptomatic improvement but inconsistent objective inflammatory outcomes and limited controlled data in UC. Across interventions, symptomatic improvement has not reliably parallelled objective inflammatory response, particularly in less controlled diets, whereas EEN and CDED show the most consistent concordance between clinical, biochemical, and mucosal outcomes. Overall, EEN and CDED (±PEN) represent the most strongly supported interventions; MD, CD-TREAT and T&H diet are promising but preliminary; and the remaining restrictive diets discussed remain investigational.

Dietary interventions must also be considered in the context of safety, feasibility and nutritional adequacy. Disease phenotype and activity influence dietary intervention suitability; for example, high-fibre dietary approaches should be undertaken with caution, and often avoided in patients with stricturing CD. Additionally, several interventions reviewed herein involve varying degrees of dietary restriction, which may increase the risk of inadequate energy intake, micronutrient deficiencies, weight loss, food-related anxiety and reduced long-term adherence. These considerations are particularly relevant in patients with active disease, baseline nutritional compromise, and pediatric populations. Psychosocial aspects of dietary therapy, including food-related quality of life (FRQoL), also warrant careful consideration. Although associations between FRQoL, eating behaviours, dietary intake, and nutritional status remain incompletely understood in IBD, impaired FRQoL is common in patients with IBD, and maladaptive eating behaviours may evolve into excessive dietary restriction and disordered eating [117]. In light of these considerations, dietary therapies should ideally be implemented within a multidisciplinary care model that includes nutritional monitoring and, where available, support from a registered dietitian experienced in IBD to ensure nutritional adequacy and facilitate safe, sustainable implementation. Given the high baseline risk of malnutrition in IBD, nutritional assessment at baseline and regularly throughout follow-up is recommended for patients undertaking restrictive dietary interventions to identify and prevent nutritional deficiencies.

## 14. Diet–Microbial Metabolism–Immune Interactions in IBD

The gut microbiome is increasingly recognized as an important mediator between diet and intestinal inflammation. Importantly, the gut microbiome comprises a complex multi-kingdom ecosystem that includes bacteria, fungi, viruses, archaea and protozoa, with alterations in bacterial, fungal and viral communities most consistently reported in IBD [118]. Although non-bacterial components remain less well-characterized than bacterial communities, they have been implicated in IBD pathogenesis and may also influence responses to dietary interventions.

Dietary exposures shape both composition and function of the gut microbiome, while microbial metabolites contribute to intestinal homeostasis by maintaining epithelial barrier integrity and mucosal immunoregulation [119,120]. Intestinal inflammation can further modify microbial composition and function, which may perpetuate dysbiosis and the inflammatory response [119]. In the setting of dysbiosis and inflammation, nutrient utilization by host cells and the gut microbiome may be altered, leading to perturbations in diet–microbiome interactions that are increasingly recognized in patients with IBD. These disruptions may contribute to inter-individual variability in responses to dietary interventions in IBD. Given these complex and interconnected relationships, it remains difficult to determine whether microbial changes observed with dietary interventions are causative drivers of clinical outcomes or simply reflect dietary exposure, underlying disease activity, or both.

Among the most well-characterized microbial metabolites in IBD are SCFAs—acetate, propionate, and butyrate—which are generated through bacterial fermentation of dietary fibre [119,121]. SCFAs support intestinal homeostasis via maintenance of epithelial barrier integrity, regulation of mucus production, and modulation of immune response [121,122]. Butyrate in particular serves as the primary energy source for colonocytes and promotes epithelial barrier function by upregulating tight junction proteins and stimulating mucin production [121,123]. It also carries anti-inflammatory effects by suppressing expression of pro-inflammatory cytokines and genes involved in inflammatory signalling pathways [121]. A key mechanism involves the activation of G-protein-coupled receptors (GPR41, GPR43, and GPR109A) expressed on immune and epithelial cells, which promote regulatory T-cell (Treg) differentiation, enhance anti-inflammatory cytokine production such as IL-18, and maintain Th17/Treg balance. Butyrate also inhibits histone deacetylase, leading to suppression of crucial pro-inflammatory mediators (including TNF-α, IL-1β, IL-2, IL-8, and IL-23), reduced expression of adhesion molecules involved in leukocyte recruitment (including TLR4, ICAM and VCAM-1), and downregulation of key inflammatory pathways such as NF-κB and IFN-γ/STAT signalling. Butyrate’s effects are further complemented by its bolstering of antioxidant defences including antioxidant enzymes, collectively reinforcing epithelial integrity and intestinal homeostasis.

Microbial production and host utilization of SCFAs appear to be impaired in IBD. Both the reduced abundance of butyrate-producing taxa, including *Roseburia hominis* and *Faecalibacterium prausnitzii*, and the impaired epithelial uptake and oxidation of butyrate due to reduced transporter expression in inflamed intestinal mucosa have been reported in patients IBD [119]. Together, these alterations may limit SCFA production and physiologic effects, potentially contributing to the inconsistent clinical efficacy of fibre supplementation and SCFA-based interventions in IBD. These findings suggest that therapeutic responses may depend not only on SCFA delivery but also on the metabolic capacity of the gut microbiome and intestinal epithelium to utilize these metabolites.

Alterations in bile acid metabolism have also emerged as important regulators of intestinal homeostasis and inflammation. Dysbiosis in IBD has been associated with impaired microbial conversion of primary to secondary bile acids, resulting in increased primary bile acids and reduced levels of secondary bile acids, such as deoxycholic acid and lithocholic acid [124]. These changes may have important functional consequences, as bile acids influence epithelial barrier integrity and immune responses through activation of bile acid receptors, including farnesoid X receptor and Takeda G-protein-coupled receptor 5 [118,125]. Recent data further suggest that microbial modulation of bile acid transport can influence host–microbe interactions relevant to intestinal inflammation in CD, highlighting a functional link between bile acid metabolism and host immune responses [126].

Diet influences both the composition of the bile acid pool and its microbial modification, and emerging evidence suggests that these changes can affect T cell responses [119]. For example, diets rich in refined ingredients have been associated with reduced levels of certain secondary bile acids and RORγ+ Treg cells, whereas supplementation with specific bile acids can restore these immune cells and attenuate intestinal inflammation in a mouse model [127]. Dietary fat may be particularly relevant, as high saturated fat intake alters bile acid conjugation and promotes the expansion of sulfite-reducing bacteria, including *Bilophila wadsworthia*, contributing to intestinal inflammation in mouse models [119,128]. Although human data regarding effects of dietary fat in IBD remain heterogeneous, these findings collectively implicate bile acid metabolism as a potential mechanism linking diet, the gut microbiome, and immunoregulation in IBD.

N-3 PUFAs, including eicosapentaenoic acid and docosahexaenoic acid, may exert anti-inflammatory effects through alterations in eicosanoid production and suppression of pro-inflammatory signalling pathways, such as NF-κB, resulting in reduced production of pro-inflammatory cytokines, including TNF-α and IL-6 [129].

In addition to shaping microbial metabolism, diet can influence intestinal inflammation through modulation of oxidative stress. Oxidative stress is increasingly recognized as a key contributor to IBD pathogenesis, promoting epithelial damage, immune activation, and chronic mucosal inflammation [130]. Emerging evidence from studies of chronic inflammatory diseases suggests that dietary patterns rich in anti-inflammatory and antioxidant nutrients may attenuate these processes by reducing reactive oxygen species (ROS) generation and modulating inflammatory pathways [129]. These dietary components include fibre and *n*-3 PUFAs, as well as other constituents such as polyphenols and antioxidant vitamins, which have attracted particular interest.

Polyphenols such as resveratrol, hydroxytyrosol, catechins, and quercetin modulate pathways involved in inflammation and cellular stress responses, including NF-κB, AP-1, and Nrf2, thereby reducing the production of pro-inflammatory mediators and oxidative damage [129]. Importantly, beyond their direct effects on host cells, polyphenols are extensively metabolized by the gut microbiome, generating bioactive derivatives that may further contribute to intestinal immunoregulation and barrier function. Similarly, antioxidant vitamins support intestinal homeostasis by enhancing endogenous antioxidant defences and limiting oxidative damage. Vitamin C scavenges ROS, thereby reducing oxidative cellular damage, whereas vitamin E protects cell membranes from oxidative damage by inhibiting lipid peroxidation [130]. Together, antioxidant nutrients help preserve epithelial integrity and blunt inflammatory signalling cascades.

Collectively, these findings reflect a growing shift from compositional descriptions of the microbiome towards a functional understanding of how diet influences microbial metabolism, host signalling pathways and intestinal inflammation [119]. However, the directionality of many observed associations remains unclear, as alterations in the microbiome and metabolome may reflect dietary exposures, disease activity, or both. Mechanistic and longitudinal studies are therefore needed to disentangle these relationships and identify causal microbiome-mediated pathways that may serve as targets for dietary intervention in IBD.

## 15. Summary and Future Directions

Dietary intervention is an attractive therapeutic option in IBD, offering the potential to supplement or, in some cases, limit medical therapy and treatment-related adverse effects and costs. Importantly, dietary therapy may also enhance patient self-efficacy in disease management. As a result, there has been increasing interest in the therapeutic potential of diet in the management and prevention of IBD, as well as the mechanistic pathways through which diet influences disease activity. However, it remains unclear whether observed microbial changes mediate the therapeutic effects of dietary interventions or instead arise secondary to improved disease activity [34]. Well-designed longitudinal studies that integrate dietary assessment with microbial, metabolomic, immunological, and clinical phenotyping are needed to better understand the complex interactions between diet, microbiome, and host immunoregulation. This would also help define the role of dietary therapy within the broader treatment paradigm of IBD, as it remains unclear whether dietary therapy would be best positioned as adjunctive to pharmacotherapy or as a standalone intervention in selected patient populations. Although RCTs remain the gold standard for informing evidence-based care, their application to dietary interventions is constrained by challenges related to adherence, feasibility, scalability, and cost [6]. Future work should also investigate potential synergistic interactions between dietary interventions and biologic and small-molecule medical therapies in optimizing mucosal healing in more severe disease.

Such uncertainties in mechanism, clinical positioningand treatment response are likely compounded by substantial variability in gut microbial composition and function across individuals, and over time within individuals, driven by environmental and host factors [131]. This heterogeneity likely contributes to the variable responses observed with dietary interventions in IBD and highlights the limitations of a uniform dietary approach. Instead, these observations support the concept that dietary therapies may be most effective when targeted to selected patient subgroups.

In this context, precision nutrition has emerged as a promising framework for advancing dietary therapy in IBD. By addressing this heterogeneity through integration of clinical characteristics with microbiome, metabolomic, immunological, and host genetic factors, precision nutrition approaches seek to identify and characterize responder and non-responder phenotypes and to develop individualized dietary interventions based on underlying biological mechanisms. Future research should focus on validating predictive models; defining causal pathways interconnecting diet, the microbiome and intestinal inflammation; and determining long-term safety, durability and efficacy of dietary interventions through large, adequately powered multicentre RCTs evaluating both induction and maintenance of remission. These studies should include longer follow-up, prespecified endpoints, diverse patient populations that reflect the heterogeneity of IBD, and standardized methods for dietary assessment and adherence monitoring. While traditional tools such as food diaries, 24 h dietary recalls and food frequency questionnaires remain valuable, dietary biomarkers and digital health technologies -including mobile applications, photographic food records, wearable devices and other passive sensing technologies -may enable more objective assessment of dietary intake, adherence, symptoms and disease activity.

Collectively, these advances may facilitate more rigorous evaluation of dietary interventions, improve understanding of treatment response and help elucidate the mechanisms linking diet, the microbiome and intestinal inflammation. Ultimately, integrating multi-omics-informed dietary interventions into personalized treatment algorithms may represent an important step towards more precise and effective management of IBD.

## Figures and Tables

**Table 1:** Dietary interventions in IBD—mechanisms, compositions, and limitations.

Diet	Proposed Mechanism	Diet Composition	Limitations of Diet
EEN	Includes anti-inflammatory properties by limiting food allergens, avoiding detrimental components and including anti-inflammatory lipid profile; influences host immune response; modulates gut microbiome	Formula provides 100% of nutritional requirements. Formula can be elemental, semi-elemental, or polymeric.	Monotony, poor palatability and the strict adherence required may compromise compliance
PEN	Same as EEN, with PEN designed to increase compliance for longer-term (maintenance) use	Combination of oral diet and formula. Oral diet can be exclusionary or unrestricted. Formula generally provides 30–50% of caloric intake.	Potential to be restrictive and difficult to maintain long term; must balance oral diet restrictions with compliance and outcomes
CDED	Emphasizes fresh whole foods hypothesized to optimize gut integrity; excludes foods hypothesized to adversely affect the gut microbiome, intestinal permeability and mucosal immune response	A 3-phase diet with PEN providing 50% to 25% of total caloric intake. Prioritizes fresh whole foods and 18–20 g of fibre daily; excludes gluten, dairy, processed foods, and food additives	Induction phase is restrictive and may affect nutritional intake
CD-TREAT	Same as EEN but uses ordinary foods to enhance tolerability	Excludes certain dietary components such as gluten, lactose and alcohol; minimizes complex carbohydrates, particularly those high in fibre; emphasizes proteins and healthy fats; micronutrient supplementation with a multivitamin	Includes processed foods to mimic EEN; can be restrictive and monotonous; strict adherence required
Tasty & Healthy diet	Excludes pro-inflammatory dietary components	A dietary framework that eliminates pro-inflammatory foods, such as processed foods, gluten, red meat, and dairy, except plain yogurt	Lack of mandatory food groups and exclusion of others may increase risk of nutritional deficiencies
SCD	Excludes poorly absorbed disaccharides and polysaccharides hypothesized to promote dysbiosis and excess organic acid production, contributing to inflammation	Limits carbohydrate intake by excluding disaccharides and most polysaccharides (such as grains, starches, most sugars and most dairy), and permitting monosaccharide-predominant foods (such as fruits and honey); encourages fresh produce, unprocessed proteins (plant-based, lean meats, poultry, fish, eggs and homemade yogurt) and healthy fats; excludes processed foods	Restrictive diet requiring strict adherence; micronutrient deficiencies have been reported
IBD-AID	Cultivates microbial homeostasis by limiting carbohydrates believed to stimulate pro-inflammatory taxa and promoting pre- and probiotic-containing foods; includes an anti-inflammatory lipid profile; modifies food textures as needed to optimize micronutrient absorption	Emphasizes pre- and probiotic foods, and omega-3 fatty acids; excludes trans fats and certain carbohydrates (lactose, sucrose, gluten-based grains and corn)	Restrictive diet; requires careful planning to ensure nutritional adequacy; requires technical ability to modify food textures as needed
AIP	Eliminates foods that potentially act as antigens that trigger immune response and indirectly disrupt the gut microbiome	Initial phase eliminates refined sugars, food additives, grains, nuts, legumes, nightshades, seeds and seed oils, dairy, eggs, coffee and alcohol; encourages fresh foods, bone broth and fermented foods. A subsequent maintenance phase gradually re-introduces food groups.	Elimination phase is restrictive, which may compromise compliance
Low-sulfur diet	Minimizes sulfur intake to reduce hydrogen sulfide and nitric oxide production, which are associated with mitochondrial dysfunction, reduced butyrate uptake by colonocytes, and reactive oxygen species over-production	Low-protein diet that limits sulfur-rich foods, especially animal proteins and products, while encouraging low-protein plant-based foods. A secondary parameter is high consumption of fermentable fibre.	Restrictive, can lead to potential nutrient deficiencies; long-term compliance rates are unclear
UCED	Avoids dietary components that adversely influence goblet cells, increase gut permeability and induce dysbiosis	A low protein, low fat (specifically animal fats and saturated fats) diet that restricts gluten, dairy, certain fermentable carbohydrates, and processed foods; emphasizes fibre, including certain fruits and vegetables, and monounsaturated fats. There is an initial 6-week elimination phase that includes mandatory foods; responders progress to a 6-week maintenance phase with gradual food reintroduction.	Rigid schedule; restricted regimen
MD	A plant-forward diet that incorporates anti-inflammatory properties, such as a beneficial lipid profile, antioxidants and fibre-driven short-chain fatty acids; enhances microbial diversity; minimizes pro-inflammatory food groups	Prioritizes fruits, vegetables, whole grains, nuts, legumes and unsaturated fats; poultry, fish, red meat and dairy allowed in moderation	Cost and accessibility can be challenging; can be difficult to tolerate in active disease

EEN = exclusive enteral nutrition; PEN = partial enteral nutrition; CDED = Crohn’s disease exclusion diet; CD-TREAT = Crohn’s disease treatment-with-eating diet; SCD = specific carbohydrate diet; IBD-AID = IBD-Anti-Inflammatory Diet; AIP = autoimmune protocol; UCED = ulcerative colitis exclusion diet; MD = Mediterranean diet.

**Table 2:** Dietary interventions in IBD—clinical evidence.

Diet	Study Design	Clinical Outcomes	Limitations of Studies	Interpretation of Evidence
EEN	RCTs, prospective cohort study, systematic reviews, meta-analyses	Remission rates of 80–85% in pediatric CD; equivalent or superior to corticosteroids in inducing clinical remission, with mucosal healing in smaller studies. Adult CD studies demonstrated inferiority to steroids in inducing clinical remission, attributed to high discontinuation rates. Limited data in UC	Heterogeneous study design and patient populations; high discontinuation rates in adult studies	More supported within the current literature
PEN	RCTs, prospective cohort studies, systematic review, meta-analysis; studies were conducted for both induction and maintenance of remission	Induction: variable effect of clinical and mucosal response; efficacy correlated with degree of oral diet restriction Maintenance of remission: increased efficacy in studies providing formula for >35% of total caloric requirements	Heterogeneity in study design and outcomes; lack of standardized definition of PEN in terms of composition and caloric requirements of oral diet	Limited/preliminary evidence
CDED (±PEN)	RCT, 2 open-label pilot studies, systematic reviews	Remission rates of 70–75% in studies of pediatric CD. RCT in pediatric CD demonstrated CDED to be as effective as EEN for induction of remission (at 6 weeks), superior for sustained remission (at 12 weeks) [24]. RCTs in adult CD demonstrated efficacy for induction and maintenance of remission, with mucosal healing in some participants.	Heterogeneity in study design (most non-randomized); most studies lack a control group; small sample sizes; limited long-term follow-up data	More supported within the current literature
CD-TREAT	RCT in healthy adult controls, open-label trial in pediatric CD (*n* = 5) [25]	Clinical response in 4/5 (80%); clinical remission in 3/5 (60%); reduction in FCP.	Small sample size; short study duration; lack of a control group in open-label trial.	Emerging evidence
Tasty & Healthy diet	RCT [26]	RCT in pediatric CD demonstrated Tasty & Healthy to be as effective as EEN in reducing FCP, ESR- and CRP; ITT analysis demonstrated higher rates of mucosal healing per MINI index in Tasty & Healthy group (54%).	Short study duration	Emerging evidence
SCD	Retrospective studies, prospective studies, multi-omics pilot RCT, RCT comparing SCD and Mediterranean diet	Sustained symptomatic improvement; transient mucosal healing (decrease in Lewis score at 12 weeks, not sustained at 1 year) [27]; variable effect on FCP	Predominantly retrospective studies and uncontrolled trials, with small sample sizes	Limited/preliminary evidence
IBD-AID	Case series, single-arm intervention pilot trial	Symptom improvement; de-escalation in medical therapy	Small sample sizes; lack of randomization and controls	Limited/preliminary evidence
AIP	Single-arm prospective study [28]	Significant improvement in symptoms; reduction in FCP that did not reach statistical significance. In total, 7/15 participants underwent endoscopic evaluation at 11 weeks, the majority of whom demonstrated endoscopic response.	Small sample size; lack of randomization and controls; short study duration	Limited/preliminary evidence
Low-sulfur diet	Case series, case reports, open-label feasibility study, open-label RCT	Symptom improvement; variable effect on FCP	Small sample sizes; short study durations; lack of standardized diet protocol, including defined sulfur threshold—accurate measurement of sulfur is challenging.	Limited/preliminary evidence
UCED	Open-label pilot study, RCT	Symptom improvement; reduction in FCP that did not reach statistical significance	Premature study closure of RCT; small sample sizes; lack of control groups; short study durations	Limited/preliminary evidence
MD	RCTs, prospective interventional study, cross-sectional study	Symptom improvement; moderate reduction in FCP and circulating markers of inflammation	Inconsistent diet definitions and compositions; fairly small sample sizes; several studies lacked controls; several studies included participants without elevated baseline FCP	Emerging evidence

EEN = exclusive enteral nutrition; PEN = partial enteral nutrition; CDED = Crohn’s disease exclusion diet; CD-TREAT = Crohn’s disease treatment-with-eating diet; SCD = specific carbohydrate diet; IBD-AID = IBD-Anti-Inflammatory Diet; AIP = autoimmune protocol; UCED = ulcerative colitis exclusion diet; MD = Mediterranean diet; RCT = randomized controlled trial; CD = Crohn’s disease; UC = ulcerative colitis; FCP = fecal calprotectin; ESR = erythrocyte sedimentation rate; CRP = C-reactive protein; ITT = intention-to-treat; MINI = Mucosal Inflammation Noninvasive index.

**Table 3:** Microbial changes associated with dietary interventions in IBD.

Diet	Taxa Increased with Diet	Taxa Decreased with Diet	Other Changes
EEN [39,40,41,42,43]	Firmicutes (p): Christensenellaceae (f) Ruminococcaceae (f) *Lactococcus* (g)	Actinobacteria (p): *Bifidobacterium* (g) Firmicutes (p): *Ruminococcus* (g) *Faecalibacterium* (g) *Faecalibacterium prausnitzii* (s) Bacteroidetes (p): Bacteroidaceae (f) Porphyromonadaceae (f) Rikenellaceae (f) *Bacteroides* (g) *Prevotella* (g)	- Increased fecal sulfide - Decreased fecal butyrate - Increase in genes encoding for spermidine/putrescine and the shikimate pathway - Decrease in genes involved in the biosynthesis of B-complex vitamins biotin and thiamine
PEN [44]	No available data	No available data	Upregulation of phosphatidylcholines.
CDED [24]	Firmicutes (p): Clostridiales (c) *Roseburia* (g) *Oscillibacter* (g) *Anaerotruncus* (g) *Ruminococcus* (g)	Actinobacteria (p): *Bifidobacterium* (g) Proteobacteria (p): Gammaproteobacteria (c) *Haemophilus* (g) Firmicutes (p): *Veillonella* (g) *Anaerostipes* (g) Bacteroidetes (p): *Prevotella* (g)	No available data
SCD [45]	Firmicutes (p): *Blautia* (s) Lachnospiraceae (f) *Faecalibacterium* (g) *Roseburia* (g) *Anaerobutyricum hallii* (s) *Eubacterium eligens* (s)	Proteobacteria (p): *Escherichia coli* (s) Firmicutes (p): *Faecalibacterium prausnitzii* (s)	No available data
IBD-AID [46]	Firmicutes (p): Clostridia (c) *Roseburia hominis* (s) *Eubacterium eligens* (s) *Faecalibacterium prausnitzii* (s) *Blautia obeum* (s) Bacteroidetes (p): Bacteroidia (c) *Bacteroides dorei* (s) *Bacteroides vulgatus* (s) *Alistipes shahii* (s)	Firmicutes (p): Clostridia (c) *Veillonella parvula* (s) *Flavonifractor plautii* (s) Bacteroidetes (p): Bacteroidia (c) *Parabacteroides distasonis* (s) *Bacteroides stercoris* (s) *Bacteroides xylanisolvens* (s) Actinobacteria (p): Coriobacteriia (c) *Collinsella stercoris* (s) *Collinsella intestinalis* (s) *Collinsella aerofaciens* (s)	
Low-sulfur diet [47,48]	No available data	No available data	- Increased butyrate, acetate, propionate, and total SCFA - Decreased BCFA:SCFA ratio - Decreased glycochenodeoxycholic acid
MD [49,50]	Firmicutes (p): Clostridia (c) *Faecalibacterium* (g) *Blautia* (g) *Ruminococcus bromii* (s) *Flavonifractor plautii* (s) *Clostridium boltae* (s) *Lactococcus lactis* (s) Bacteroidetes (p): *Alistipes finegoldii* (s)	Firmicutes (p): *Veillonella dispar* (s) *Veillonella tobetsuensis* (s) *Streptococcus australis* (s) *Massilioclostridium methylpentosum* (s) *Blautia hydrogenotrophica* (s) Bacteroidetes (p): *Prevotella copri* (s) Actinobacteria (p): *Bifidobacterium animalis* (s) Proteobacteria (p): *Escherichia*/*Shigella* (g)	Increased butyrate, acetate, valerate, and total SCFA

EEN = exclusive enteral nutrition; PEN = partial enteral nutrition; CDED = Crohn’s disease exclusion diet; SCD = specific carbohydrate diet; IBD-AID = IBD Anti-Inflammatory Diet; MD = Mediterranean diet; p = phylum; c = class; f = family; g = genus; s = species; SCFA = short-chain fatty acid; BCFA = branched-chain fatty acid. Reported taxonomic shifts should be interpreted in the context of heterogeneous study designs and methodologies.

## Data Availability

No new data were created or analyzed in this study. Data sharing is not applicable to this article.
